# Reversibly Alterable Hot-Electron Photodetection Without Altering Working Wavelengths Through Phase-Change Material Sb_2_S_3_

**DOI:** 10.3390/mi16020146

**Published:** 2025-01-26

**Authors:** Yaoyao Li, Xiaoyan Yang, Jia Hao, Junhui Hu, Qingjia Zhou, Weijia Shao

**Affiliations:** 1 School of Physical Science and Technology & Guangxi Key Laboratory of Nuclear Physics and Technology, Guangxi Normal University, Guilin 541004, China; yaoyaoli@stu.gxnu.edu.cn (Y.L.); jiahao@stu.gxnu.edu.cn (J.H.); hujh@mailbox.gxnu.edu.cn (J.H.); 2 University Engineering Research Center of Advanced Functional Materials and Intelligent Sensing, Guilin 541004, China; 3 School of Politics and Public Administration, Guangxi Normal University, Guilin 541004, China; xyyang@gxnu.edu.cn

**Keywords:** phase-change material, photodetectors, hot electrons, working wavelengths

## Abstract

Generally, the responsivities of hot-electron photodetectors (HE PDs) are mainly dependent on the device working wavelengths. Therefore, a common approach to altering device responsivities is to change the working wavelengths. Another strategy for manipulating electrical performances of HE PDs is to harness electric bias that can be used to regulate hot-electron harvesting at specified working wavelengths. However, the reliance on bias hampers the flexibility in device operations. In this study, we propose a purely planar design of HE PDs that contains the phase-change material Sb_2_S_3_, realizing reversibly alterable hot-electron photodetection without altering the working wavelengths. Optical simulations show that the designed device exhibits strong absorptance (>0.95) at the identical resonance wavelengths due to the excitations of Tamm plasmons (TPs), regardless of Sb_2_S_3_ phases. Detailed electrical calculations demonstrate that, by inducing Sb_2_S_3_ transitions between crystalline and amorphous phases back and forth, the device responsivities at TP wavelengths can be reversibly altered between 59.9 nA/mW to 128.7 nA/mW. Moreover, when device structural parameters are variable and biases are involved, the reversibly alterable hot-electron photodetection at specified TP wavelengths is maintained.

## 1. Introduction

Extracting energetic carriers (e.g., hot electrons) generated in metals has been shown to provide promising benefits to a number of photonic technologies, such as photovoltaics [1,2], photocatalysis [3,4], and photodetection [5,6]. In particular, despite extremely low internal quantum efficiencies (IQEs), hot-electron photodetection has been attracting great attention due to its salient capabilities of room-temperature operation [7], gap-free detection [8], and ultra-short responding time [9]. In practice, in order to generate abundant populations of hot electrons to be harvested further, various nanostructured hot-electron harvesting junctions have been explored to strongly capture massive photons in metallic materials through the resonant light–matter interactions [10,11,12,13,14,15,16]. However, nanostructured hot-electron photodetectors (HE PDs) often require complicated/costly fabrications and can hardly overcome the difficulties in large-scale applications [17]. In contrast, with the aid of optical excitations in multi-layer thin film stacks, including Fabry–Pérot resonances [17,18,19] and Tamm plasmons (TPs) [20,21,22], planar HE PDs exhibit clear advantages in cost-effective fabrication and large-area applications.

Over the past decade, several HE PDs with different planar architectures have been deeply studied to achieve two primary objectives, namely, customized spectral responses and enhanced IQEs, as briefly presented in the following. Motivated by realizing planar broadband hot-electron photoelectric conversion, high refractive index absorbable materials, such as Cr [23], Pt [24], Ti [25], and TiN [26], have been employed to efficiently capture light energies over a wide wavelength range. Recently, it has been demonstrated that, when planar metal–dielectric-metal junctions were inserted between two distributed Bragg reflectors (DBRs), HE PDs exhibited ultranarrow responsivity bandwidths as low as 0.4 nm due to the excitations of coupled dual TPs [27]. On the other hand, several strategies aimed at boosting device IQEs by fundamentally manipulating hot-electron dynamics have been exploited, including the engineering of the electronic density of states of metals to raise the proportions of above-barrier hot electrons [28,29], the adoptions of ultrathin absorbable film to suppress hot-electron transport losses [30,31], and multi-junction designs to increase hot-electron collection paths [32,33].

In spite of the aforementioned progresses, reversibly altering the electrical outputs of planar HE PDs at specified working wavelengths is still a challenge. Basically, the responsivities of HE PDs are primarily determined by the device working wavelengths through two factors. One is the wavelength-dependent absorption efficiency in metals. Planar HE PDs often operate at the wavelengths associated with maximum absorptance in metals, guaranteeing to harvest as many photoexcited hot electrons as possible. The other is the wavelength-dependent IQE. The negative correlation between working wavelengths and IQEs can be described by the well-known Fowler equation, imposing limitations on device electrical performances even with perfect absorptions [10]. Therefore, for those planar HE PDs that have already been fabricated, the absorption spectra are determinate and the responsivities at specified operating wavelengths are unalterable. In practice, external electrical voltages applied to hot-electron harvesting junctions can be used to regulate the hot-electron collection efficiencies, resulting in continuously variable responsivities at specified working wavelengths. However, the reliance on electrical bias may reduce the flexibility in device operations and probably lead to an unfavorable increase in dark currents [11]. In consequence, an alternative approach to realizing alterable and reversible planar hot-electron photoelectric conversion without altering working wavelengths and applying bias is highly desired.

The phase-change material Sb_2_S_3_ has been used to alter the resonance wavelengths of HE PDs due to its salient capabilities of an ultra-short switching time (~70 ns), phase stability at room temperature, and large bandgap [34,35]. In this work, we demonstrated a Sb_2_S_3_-involved route to reversibly alter device responsivity at specified working wavelengths through changing Sb_2_S_3_ phases. We designed a planar TP-based HE PD in which the barrier heights of hot-electron harvesting junctions are determined by Sb_2_S_3_ phases. Optical simulations revealed that the TP wavelength (945 nm) and TP-induced strong absorption (>0.95) are independent of the Sb_2_S_3_ phases, albeit with obvious distinctions in the refractive indexes of two different Sb_2_S_3_ phases. In contrast, electrical calculations show that the device responsivities at 945 nm can be reversibly altered between 59.9 nA/mW and 128.7 nA/mW by inducing Sb_2_S_3_ phase transitions back and forth. When variable structural parameters and the effects of electric bias are taken into account, the designed device has strong robustness in reversibly alterable photoelectric conversion. Our study forecasts more degrees of freedom in the operation of hot-electron devices.

## 2. Results and Discussion

**Figure 1:**
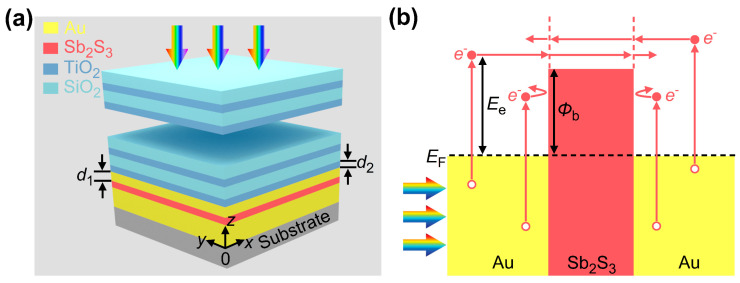
(**a**) Schematic of the designed device. (**b**) Energy diagram of Au-Sb_2_S_3_-Au junction for hot-electron harvesting.

As schematically shown in Figure 1a, the designed HE PD consists of a planar Au-Sb_2_S_3_-Au junction and a DBR that is composed of eight pairs of alternatively arranged TiO_2_ and SiO_2_ layers. The designed device is mounted on a silica substrate. Both Au layers serve as absorbers and electrodes simultaneously. The electrode intermediate layer is made of Sb_2_S_3_ that possesses crystalline (Cry) and amorphous (Amp) phases between which suitable opto-thermal or electro-thermal treatments can be carried out to induce transitions reversibly [34]. The bottom Au layer has a thickness of 100 nm, and the Sb_2_S_3_ layer has a thickness of 5 nm. The thickness of the top Au layer is denoted by *d*_1_. The TiO_2_ layer, which is adjacent to the top Au layer, is denoted by *d*_2_. Unless specifically indicated, the thicknesses of TiO_2_ and SiO_2_ layers in the DBR are 105 nm and 155 nm, respectively. Figure 1b displays that the operation of the designed device relies on three electronic processes within the Au-Sb_2_S_3_-Au junction, i.e., hot-electron generation, transport, and collection. Specifically, the energy depositions of incident light in the two Au layers result in the electronic transitions from occupied levels below the Fermi level (*E*_F_) to higher unoccupied levels, generating non-equilibrium electrons whose energy distributions deviate substantially from equilibrium Fermi–Dirac distributions [8]. Thus, these energetic electrons would experience ultrafast thermalizations within dozens of femtoseconds and diffuse to Au-Sb_2_S_3_ interfaces simultaneously [36]. The transport losses are determined by the distances of hot electrons to the Au-Sb_2_S_3_ interfaces and the hot-electron energies (*E*_e_) exceeding *E*_F_. Upon arriving at the two interfaces, hot electrons would come up against the Au-Sb_2_S_3_ barrier, whose height (*Φ*_b_) is related to the phases of Sb_2_S_3_ (*Φ*_b_ = 0.57 eV for Cry phase and *Φ*_b_ = 0.35 eV for Amp phase) [35]. Among them, above-barrier (*E*_e_ > *Φ*_b_) hot electrons have a chance of entering the Sb_2_S_3_ layer and then reaching the counter electrode, accompanied by *E*_e_-dependent interfacial losses. However, below-barrier (i.e., *E*_e_ < *Φ*_b_) hot electrons would be blocked. The net hot electrons collected by the two electrodes contribute to a steady-state photocurrent with the aid of an external circuit.

**Figure 2:**
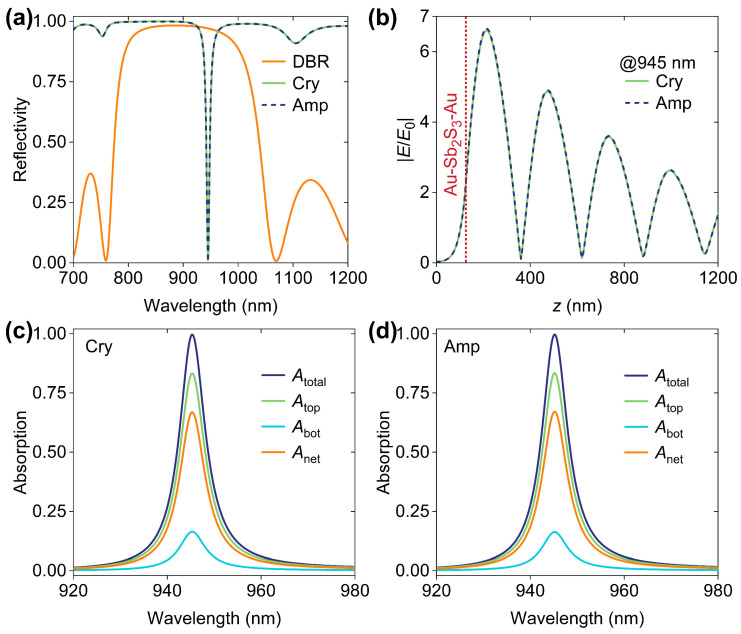
(**a**) Wavelength-dependent reflectivity of the bare DBR and the designed device for Cry- and Amp-phase Sb_2_S_3_. (**b**) Spatial distributions of normalized electric fields (|*E*/*E*_0_|) at TP wavelength of 945 nm. Wavelength-dependent absorption contributions of the designed device when Sb_2_S_3_ exhibits (**c**) Cry and (**d**) Amp phases. During calculation, *d*_1_ = 20 nm and *d*_2_ = 105 nm.

First of all, we examined the reflection spectra of the designed device with the finite element method, as shown in Figure 2a. For comparison, the reflection spectrum of a bare DBR mounted on the same silica substrate was also plotted. It was found that, for both Sb_2_S_3_ phases, there is a reflection dip at 945 nm that lies in the DBR forbidden band, indicating the excitations of TPs [7]. In other words, although the Sb_2_S_3_ refractive index difference between Cry and Amp phases is approximately 0.5 [35], the phase change of Sb_2_S_3_ has a negligible impact on the device optical responses since the Sb_2_S_3_ layer is ultrathin. To gain more insights into the two TP excitations, we investigated the spatial distributions of electric field intensity (|*E*|) at 945 nm, normalized to the electric field intensity (|*E*_0_|) of the incident light, as shown in Figure 2b. We found that, as an interfacial optical excitation, TP resonances result in prominent field enhancements (i.e., |*E*/*E*_0_| > 1) around the interface between the DBR and the Au-Sb_2_S_3_-Au junction, probably leading to energy depositions in the two absorbable Au layers. Figure 2c,d depict the wavelength-dependent absorption efficiencies in the top (*A*_top_) and bottom (*A*_bot_) Au layers when Sb_2_S_3_ exhibits the Cry and Amp phases, respectively. The spectra of total (*A*_total_) and net (*A*_net_) absorptions are also shown. *A*_total_ and *A*_net_ are expressed by *A*_total_ = *A*_top_ + *A*_bot_ and *A*_total_ = *A*_top_ − *A*_bot_, respectively. We found the following: (1) the absorption spectra for Sb_2_S_3_ in both Cry and Amp phases are nearly identical; (2) TPs induce strong absorptions in Au layers (*A*_total_ > 0.95 at 945 nm); and (3) the absorptions in the two Au layers are asymmetric (*A*_top_ > *A*_bot_ and *A*_net_ > 0).

**Figure 3:**
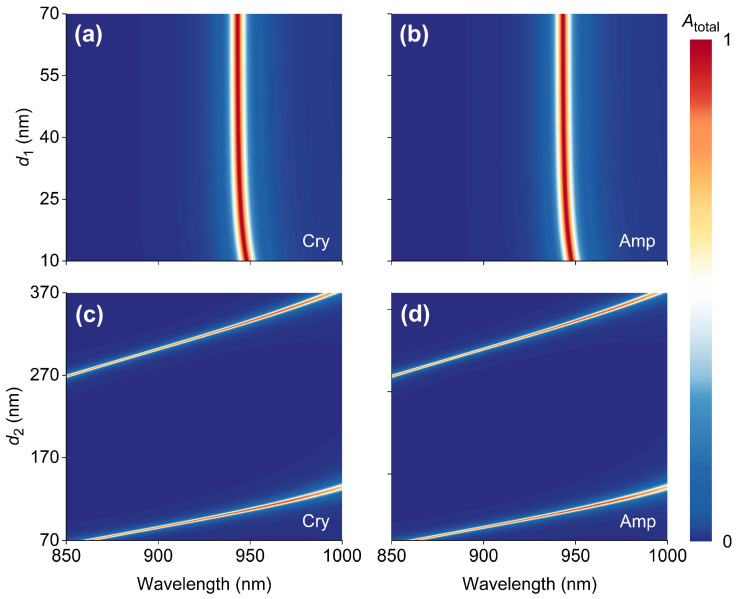
For *d*_2_ = 105 nm, the wavelength-dependent *A*_total_ as a function of *d*_1_ under the circumstances of (**a**) Sb_2_S_3_ Cry and (**b**) Amp phases are shown. For *d*_1_ = 20 nm, the contour maps of *A*_total_ when *d*_2_ increases from 70 nm to 370 nm for (**c**) Cry- and (**d**) Amp-phase Sb_2_S_3_ are shown.

To investigate the spectral tunability of the designed device, when *d*_2_ is fixed at 105 nm and *d*_1_ increases from 10 nm to 70 nm, we studied the wavelength-dependent *A*_total_ in the cases of Cry- and Amp-phase Sb_2_S_3_, as depicted in Figure 3a and Figure 3b, respectively. It was found that, for both Sb_2_S_3_ phases, the increases in *d*_1_ result in slight blueshifts of TP wavelengths and strong TP absorption efficiencies are maintained. Figure 3c,d show that, when *d*_1_ is fixed at 20 nm and *d*_2_ increases beginning from 70 nm, TP wavelengths undergo redshifts regardless of Sb_2_S_3_ phases. Moreover, two additional absorption bands appear when *d*_2_ increases further, indicating the excitations of high-order TPs. Anyway, it is convenient to tailor the optical responses by adjusting the structural parameters (*d*_1_ and *d*_2_).

**Figure 4:**
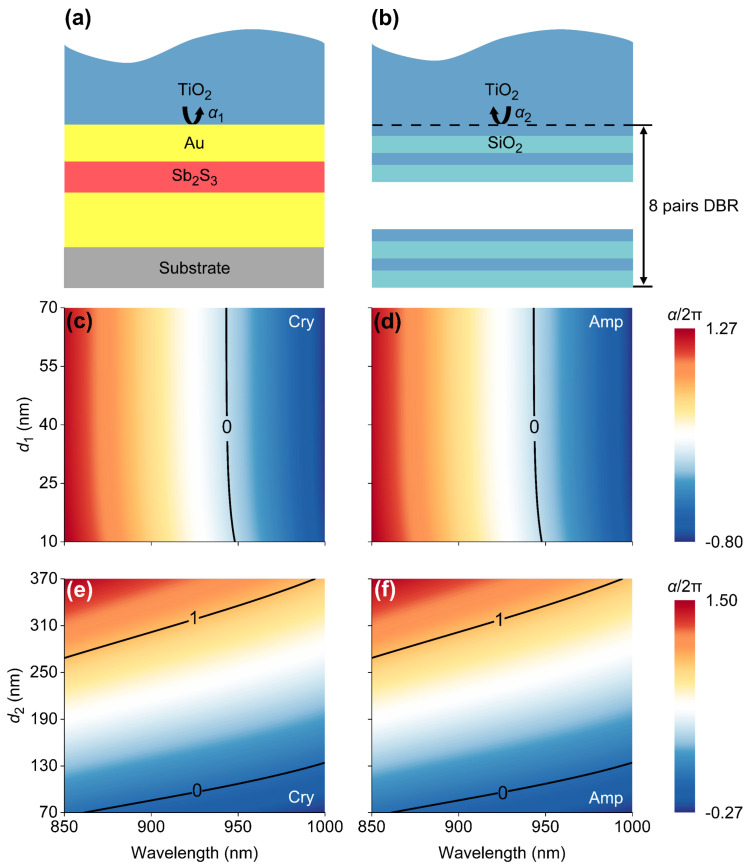
Schematics for the calculations of phase shifts, i.e., *α*_1_ and *α*_2_, due to the reflections off the (**a**) Au-Sb_2_S_3_-Au junction and (**b**) DBR in a TiO_2_ medium, respectively. Wavelength-dependent phase accumulations (*α* = *α*_1_ + *α*_2_) as a function of *d*_1_, ranging from 10 nm to 70 nm, when Sb_2_S_3_ is in the (**c**) Cry and (**d**) Amp phases. Contour maps of *α* when *d*_2_ increases from 70 nm to 370 nm for (**e**) Cry- and (**f**) Amp-phase Sb_2_S_3_.

To reveal the underlying physics governing TP excitations, we investigated the accumulation of phase shifts resulting from the reflection at the interface between the Au-Sb_2_S_3_-Au junction and the DBR. Specifically, we employed the transfer matrix method to calculate the phase shift (*α*_1_) caused by the light reflection off the DBR in a TiO_2_ medium, as shown in Figure 4a. The phase shift (*α*_2_) originating from the light reflection off the Au-Sb_2_S_3_-Au junction in a TiO_2_ medium was also calculated, as shown in Figure 4b. Based on *α*_1_ and *α*_2_, we obtained the interfacial phase accumulation (*α*), which can be expressed by *α* = *α*_1_ + *α*_2_. The wavelength-dependent *α*, normalized by 2π, is closely related to *d*_1_ and *d*_2_, as shown in Figure 4c–f, in which six contour lines represent *α* = 0 or *α* = 1. Interestingly, we found that the contour lines (*α* = 0) in Figure 4c,d are perfectly consistent with the absorption bands that are depicted in Figure 3a and Figure 3b, respectively. Similarly, when *d*_2_ increases from 70 nm to 370 nm, the absorption bands in Figure 3c and Figure 3d correspond to the contour lines that are depicted in Figure 4e and Figure 4f, respectively. In brief, we have offered quantitative evidence to suggest the phase matching condition allowing for TP excitations [26] that can be described by

(1) α=2kπ    k=0,1,2…

**Figure 5:**
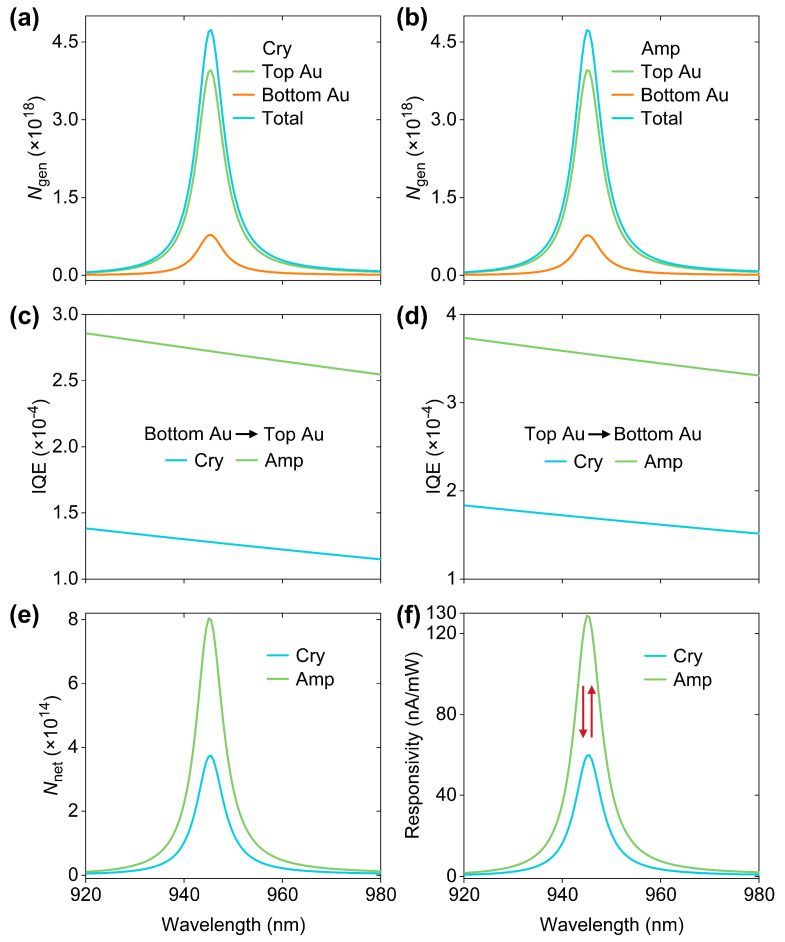
(**a**) Spectra of the fluxes (*N*_gen_) of the generated hot electrons when Sb_2_S_3_ is in the (**a**) Cry and (**b**) Amp phases. For both Sb_2_S_3_ phases, the wavelength-dependent internal quantum efficiencies (IQEs) from (**c**) bottom to top Au layers and (**d**) from top to bottom electrodes are shown. (**e**) The spectra of calculated fluxes (*N*_net_) of the net collected hot electrons and (**f**) the predicted responsivities as a function of wavelength under two circumstances of Sb_2_S_3_ Cry and Amp phases.

On the basis of sufficient optical investigations, electrical assessments of the designed HE PDs are feasible using a well-established model. In this model, the power for any wavelength of the incident light was normalized to be 1 W and the electronic processes for hot-electron harvesting were quantitatively treated [7,11]. When Sb_2_S_3_ is in the Cry and Amp phases, we calculated the wavelength-dependent fluxes (*N*_gen_) of the generated hot electrons in different Au layers, as can be seen in Figure 5a and Figure 5b, respectively. It was found that the populations of TP-induced hot-electrons per second in the top Au layer are larger than those in the bottom Au layer. This is because TP-induced absorptions in the top Au layer are larger than that in the bottom Au layer for both Sb_2_S_3_ phases. Meanwhile, the hot-electron generation rates are in direct proportion to the absorption efficiencies. By accounting for hot-electron transport losses within the Au layers and interfacial losses at the two Au-Sb_2_S_3_ interfaces, we obtained the wavelength-dependent IQEs that were defined as a ratio of the fluxes (*N*_col_) of collected hot electrons to *N*_gen_, as shown in Figure 5c,d. Evidently, the IQEs decrease with the increase in wavelength due to the reduction in proportion of above-barrier hot electrons. In addition, the IQEs from the top to bottom Au layers in the case of Amp-phase Sb_2_S_3_ are larger than that in the case of Cry-phase Sb_2_S_3_ due to the decreased barrier height. This trend is also observed when calculating the IQEs from the bottom to top Au layers. As a result, although the *N*_gen_ values at the TP wavelengths are nearly identical for both Sb_2_S_3_ phases, the Sb_2_S_3_ phase transition dramatically affects the fluxes (*N*_net_) of the net collected hot electrons, which can be calculated by subtracting the *N*_col_ of the top to bottom Au layers from the *N*_col_ of the bottom to top Au layers, as shown in Figure 5e. As expected, the *N*_net_ at 945 nm for Cry-phase Sb_2_S_3_ is less than that under the circumstance of Sb_2_S_3_ in the Amp phase. Figure 5f displays the calculated responsivity spectra for both Sb_2_S_3_ phases. It is suggested that the device responsivities at 954 nm can be reversibly altered between 59.9 nA/mW and 128.7 nA/mW by inducing Sb_2_S_3_ transition, back and forth, between the Cry and Amp phases.

**Figure 6:**
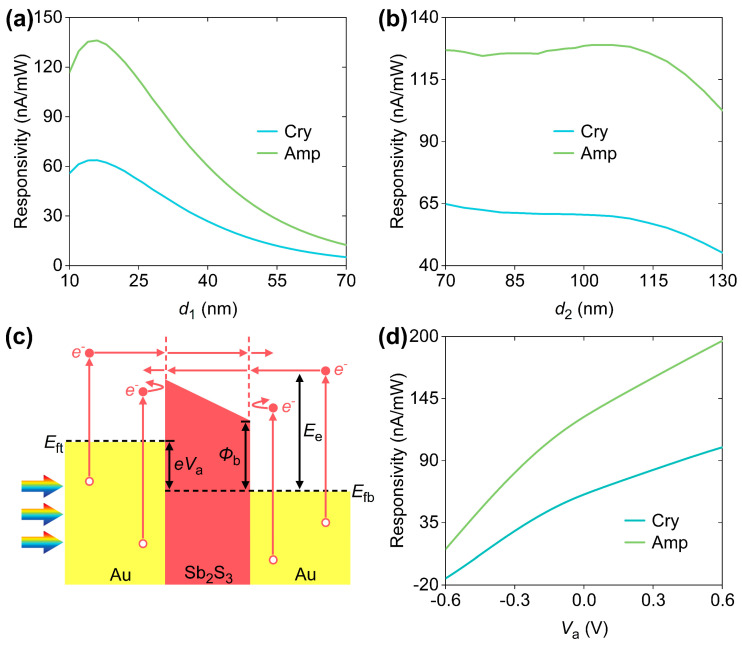
For both Sb_2_S_3_ phases, the TP-induced responsivities as a function of (**a**) *d*_1_ and (**b**) *d*_2_ are shown. *d*_2_ is fixed at 105 nm in (**a**) and *d*_1_ is fixed at 20 nm in (**b**). (**c**) The energy diagram of Au-Sb_2_S_3_-Au junction when external electrical voltage (*V*_app_) is applied between two Au electrodes. *E*_ft_ (*E*_fb_) is the Fermi level of the top (bottom) Au layer. (**d**) *V*_app_-dependent responsivities at 945 nm for different Sb_2_S_3_ phases, where *d*_1_ = 20 nm and *d*_2_ = 105 nm.

Finally, we assessed the robustness of reversibly alterable hot-electron photodetection when the structural parameters of the designed device are variable and the external electric voltage (*V*_app_) between the two Au electrodes becomes involved in hot-electron extraction. As shown in Figure 6a, for both Sb_2_S_3_ phases, the peak responsivities initially increase and then decrease as *d*_1_ increases from 10 nm to 70 nm, with *d*_2_ fixed at 105 nm. This is because the increase in *d*_1_ results in the increase in *A*_net_ at the TP wavelengths, enhancing the asymmetric hot-electron generation between the two Au layers. However, with the further increase in *d*_1_, substantial hot-electron transport losses would offset the benefits from the increase in *A*_net_. Figure 6b shows that, with *d*_1_ fixed at 20 nm, the clear distinctions in peak responsivities between Cry- and Amp-phase Sb_2_S_3_ are maintained for different TP wavelengths as *d*_2_ increases from 70 nm to 130 nm. Besides *d*_1_ and *d*_2_, electric bias (*V*_app_) can also be employed to tune the hot-electron harvesting by adjusting the effective barrier height of the Au-Sb_2_S_3_ interface, as shown in Figure 6c. Detailed calculations reveal that, at a TP wavelength of 945 nm, a positive *V*_app_ boosts device responsivity, while a negative *V*_app_ degrades electrical performances, as shown in Figure 6d. Overall, in many cases, the designed device has displayed reversibly alterable photoelectric conversion at specific working wavelengths.

## 3. Conclusions

Although it is easy to assume that the barrier heights of hot-electron harvesting junctions are tunable for alterable device responsivities, the relevant designs of HE PDs lack feasible routes [29]. In this work, we have demonstrated a planar design of TP-based HE PDs in which an ultrathin Sb_2_S_3_ layer is sandwiched between two Au layers, forming a Au-Sb_2_S_3_-Au junction. Detailed optical calculations revealed that Sb_2_S_3_ phase transitions have negligible impacts on the optical responses of the designed device from the perspectives of both the TP wavelengths and TP-induced absorption efficiencies. However, the responsivities at the TP wavelengths can be reversibly altered by inducing a phase transition between Cry- and Amp-phase Sb_2_S_3_ due to the different barrier heights of the Au-Sb_2_S_3_-Au junction. Further studies have clarified that the reversibly alterable photodetection sustains regardless of structural parameters and electric bias. It is believed that, besides electric bias, phase-change materials are feasible candidates for altering responsivities at specified working wavelengths and provide progress for the applications of infrared techniques [37,38].

## Data Availability

The raw data supporting the conclusions of this article will be made available by the authors upon request.
